# Jet and Shock Wave from Collapse of Two Cavitation Bubbles

**DOI:** 10.1038/s41598-018-37868-x

**Published:** 2019-02-04

**Authors:** Jing Luo, Zhipan Niu

**Affiliations:** 1 State Key Laboratory of Hydraulics and Mountain River Engineering, Sichuan University, Chengdu, 610065 China; 2 Institute for Disaster Management and Reconstruction, Sichuan University, Chengdu, 610207 China

## Abstract

As a common hydrodynamic phenomenon, multi-cavitation dynamics is widely found in many industries such as hydraulic engineering, shipping industry and chemical industry. The jet and shock wave phenomenon in the interaction of two cavitation bubbles are the basis of multi-cavitation bubbles interaction research. By respectively inducing two cavitation bubbles through laser and underwater low-voltage discharge, this paper tested the jet and shock wave resulting from the collapse of the two cavitation bubbles, and the following conclusions are obtained: (1) If the two cavitation bubbles are synchronously generated but in different size, as the distance between the two cavitation bubbles increases or the maximum radius of the smaller cavitation bubble increases, the effect of the small cavitation bubble on the larger one gradually changes from the surface wave phenomenon to jet that breaks through the larger bubble. When the two bubble center lines are parallel to the wall surface, this jet suppresses the formation of the jet to the wall surface when the large cavitation bubble collapses; if the two cavitation bubbles are generated at the same time with same size, as the initial distance of the two cavitation bubbles gradually decreases, the two bubbles are more likely to form a face-to-face collapse, and the smaller the distance between the two, the easier it is to fuse. (2) The impact of the initial moment of the cavitation bubble on the structure of the collapse shock wave is as follows: for two bubbles of different sizes formed synchronously, the shock wave propagates to the periphery in the form of a number of consecutive waves appearing in the larger bubble, while for the unsynchronized ones, shock waves appeared in both cavitation collapses, and a number of consecutive waves appear in the late-formed cavitation bubble. And multiple consecutive shock waves may overlap in some areas of the space. These conclusions have obvious implications for preventing cavitation damage and utilization of cavitation.

## Introduction

Cavitation is a very typical hydrodynamic phenomenon that exists in many fields, such as hydraulic engineering, shipping, ultrasonic cleaning, underwater explosions and the medical industry. Since the size of the cavitation bubble is very small (millimeters) and the life cycle is very short (milliseconds or even microseconds), it is very difficult to directly observe cavitation in the engineering field. Therefore, high-speed photography is a must for observing the jet and shock waves generated by cavitation collapses, so as to explore the internal mechanism of cavitation dynamics.

There are many reports on single cavitation bubble dynamics and the interaction between bubbles and wall surfaces. Regarding the dynamics of cavitation bubbles in the unbounded domain, Obreschkow *et al*.^1^ concluded that when *ρgR*_*max*_/*P*_*∞*_ > 4*10^−4^, the jet formed during the collapse of the cavitation bubble will pierce the far-end surface of the cavitation. Where: *ρ* is the liquid density, *g* is the gravitational acceleration, *R*_*max*_ is the radius at which the cavitation bubble develops to the maximum volume, and *P*_*∞*_ is the hydrostatic pressure of the surrounding liquid. When the cavitation bubble shrinks and collapses, the Weber number and Reynolds number are so large that the effect of liquid viscosity and surface tension on the collapse of the cavitation bubble is negligible^2–4^. It can be seen from many current research results that the direction of jets in the late stage of cavitation collapse is strongly influenced by the surrounding boundary. For the cavitation near the free surface, the cavitation is attracted by the free surface during the expansion phase. When the jets appeared in the contraction phase, the jets deviate from the free surface^5–8^. With regard to the interaction of cavitation bubbles with rigid walls, high-speed jets of cavitation bubbles in the late stages of collapse develop towards the wall^2,9–11^, this jet towards the wall is an important part of the research on the cavitation erosion mechanism^12^.

In the real field of production, cavitation bubble often do not appear in isolation. As a result, in recent years, some new research interests on cavitation were proposed, such as studies on the interaction between cavitation bubbles and cavitation bubbles, cavitation bubbles and air bubbles, cavitation bubbles and particles, and cavitation bubbles and ice block have also been reported. Pain *et al*.^13^ studied the jet flow in an air bubble induced by a nearby cavitation bubble and discovered that the speed of such a jet flow can be up to 250 m/s. Luo *et al*.^14^ used the method of high-voltage discharge technology generate a cavitation bubble to study the interaction between a cavitation bubble and an air bubble. Goh *et al*.^15^ studied the interactions between hemispheric air bubbles placed below the underwater slab and a cavitation bubble, and discovered that the ratio between oscillation time of the cavitation bubbles and the oscillation time of the air bubbles is the important parameter impacting the jet caused by cavitation bubbles collapse. The interaction between a cavitation bubble and a particle was researched by adopting a high-voltage discharge technology to induce cavitation bubble^16^ and low-voltage underwater discharge technology to induce a cavitation bubble^17^. The interaction between ice block and cavitation bubble was researched by Cui *et al*.^18^, the direction of jet and the propagation of shock waves were captured.

Regarding the cavitation clouds appearing in the actual project (such as the cavitation clouds inside the flood discharge tunnel in the water conservancy project), the interaction between the cavitation bubbles inside the cavitation cloud directly affects the destructive strength of the cavitation cloud collapse. Therefore, the study of the interaction mechanism between the cavitation bubbles inside the cavitation group is very important. If the two cavitation bubbles are very close at the time of inception, the surfaces of the two close to each other are flattened due to the extrusion of the expansion process, gradually approaching, and finally the two bubbles fuse together. The interaction of two cavitation bubbles is the most basic as well as the foremost part for the study of interactions of multiple bubbles. The fusion phenomenon is typical and complex, on which the researches are still incomplete so far. For two cavitation bubbles formed at the same time, the jets that appear in the late collapse of the cavitation develop toward each other^19–21^. However, in reality, each cavitation bubble in cavitation group has different properties, such as the time of inception, the size and the position at inception. Above all, in addition to these factors, the boundary around the bubble has a very important impact on the collapse characteristics of the bubbles. Bremond *et al*.^22^ experimentally studied the interaction of two hemispherical cavitation bubbles equivalent to two spherical ones and studied their interactions with rigid walls. Tomita *et al*.^23^ studied the interaction of two laser-induced cavitation bubbles near the wall surface. Blake *et al*.^19^ conducted a corresponding numerical simulation study on the conditions in the above literature. Robinson *et al*.^24^ studied the interaction of two cavitation bubbles under the free surface using a combination of experimental and numerical simulations, and found that if the distances between the two cavitation bubbles are very close, the overall collapse characteristics are greatly affected by the free surface. Chew *et al*.^25^ studied two cavitation bubbles under the circumstances that the two bubble center lines are parallel to the wall surface, and they obtained a jet prediction method under the dual influence of cavitation and wall surface.

In addition, there are some reports on the study of the fusion of two cavitation bubbles. Through a comprehensive comparison of theoretical and experimental studies, Bremond *et al*.^22^ found that the inertia of the liquid film between them had an important impact on fusion before the two cavitation bubbles were fused. Rungsiyaphornrat *et al*.^26^ proposed a mathematical model that can be used to simulate the interaction of two underwater explosive bubbles using the boundary integral method (BIM). Han *et al*.^27^ studied the dynamics of fused bubbles using numerical simulations. Although there have been some reports on fusion bubbles, the current research results are less concerned with the dynamics of fusion bubbles, especially the dynamics of cavitation bubbles to the ring stage. In recent years, with the rise of new computational methods, Smoothed Particle Hydrodynamics (SPH) has been gradually applied to the study of bubble dynamics. Zhang^28^ established an axisymmetric SPH numerical model for bubble dynamics, simulated the underwater explosion bubble combined with Boundary Element Method (BEM), and obtained jets and shock waves that are highly consistent with the experimental results. As the SPH computational method is gradually applied to the research field of fluid–structure interactions^29^, this new computational method will become a new way to solve complex bubble dynamics problems and bubble-wall interactions.

Based on the above-mentioned researches on two cavitation bubbles and multi-cavitation bubbles, this paper qualitatively discussed the surface wave phenomena, the development of internal jets, and the development of two bubble shock waves that occur during the interaction of two cavitation bubbles with a big difference in size. And the evolution characteristics of two cavitation bubbles induced by the two systems were compared.

## Experimental Setups

**Figure 1:**
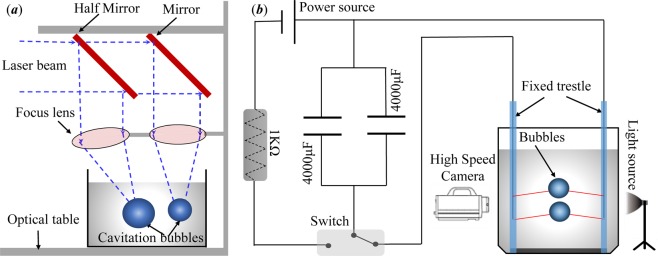
Schematic Diagram of the Experimental Device.

Studying interactions of two cavitation bubbles from the mesoscopic level requires a multi-cavitation-inducing system. The jet and shock wave phenomena appearing in the interaction between the cavitation bubbles need to be observed by means of a high-speed dynamic acquisition and analysis system. In this paper, the method of laser-induced cavitation is mainly used for the jet phenomenon in the two cavitation bubbles interactions. The principle is shown in Fig. 1a, and for the shock wave generated during the collapse of the two cavitation bubbles, the method of underwater low-voltage discharge^30^ was adopted, the principle of which is shown in Fig. 1b.

The method of using laser underwater focusing to induce cavitation bubble is shown in Fig. 1a. The use of laser is advantageous as it is highly accurate in controlling the bubble nucleation spot and it is nonintrusive to the dynamics of two cavitation bubbles. The beam emitted by the Q-switched Nd: YAG laser (wavelength 1064 nm, pulse duration 8 ns) has a diameter of 5 mm. After the laser beam is first passed through the beam expander, the beam diameter is expanded to 30 mm, and then it passes through a half-transmitting mirror and a mirror so as to change the direction of the laser beam, and to project it vertically downward to the focusing mirror (the focal length is 75 mm), and the focused beams are respectively concentrated in the water tank. By replacing the half-transmitting mirrors of different transmittances and adjusting the angles of the corresponding focusing mirrors, the relative size and relative position of the cavitation bubbles in the water can be precisely adjusted. The water tank has a size of 25 cm*20 cm*25 cm and is filled with secondary deionized water with a liquid surface height of 23 cm. The experimental environment temperature is constant at around 24 °C.

The method of inducing cavitation bubbles through low-voltage underwater discharge is shown in Fig. 1b. In the charging circuit, the current is charged into two parallel capacitors (voltage is 100 V) through the resistor. When the capacitor is fully charged, switch to the discharge circuit. In the discharge circuit, the current is instantaneously discharged through the contact electrode inside the tank. The contact point of the electrode generates a large amount of heat, and the water body near the electrode contact point is instantaneously vaporized to form a bubble (while the end of the electrode contact point is fused). The water tank size is 25 cm*20 cm*25 cm, and the tank is filled with secondary deionized water. The depth of the water is 23 cm, and the experimental environment temperature is 24 °C. In order to minimize the influence of the electrode on the evolution characteristics of the bubbles, in our experiment, a copper wire with a diameter of 0.1 mm was selected as the discharge electrode (the bubble radius generated by the experimental system is 5.00 mm to 12.00 mm).

The evolution cycle of the cavitation bubbles is very short. In order to observe the cavitation dynamics process, a high-speed dynamic acquisition and analysis system must be adopted. The system consists of a high-speed camera, a macro lens, and a light source. Among them, the experiment used Fastcam SA-Z high-speed camera (Photron Inc., Japan, the highest acquisition rate is 1,000,000 fps). Due to the small size of the cavitation bubbles, the experiment used a macro lens (Nikon, Micro 105/2.8 G) for the high-speed camera. In order to obtain a clear bubble profile, the evolution and migration of the cavitation bubbles, as well as the development of jets, a cold light source (power: 150 W) must be used as a background supplemental light source. For the evolution of shock waves during cavitation bubble collapse, we used a white light (power: 3 W) with parallel light source as a background light. The shock waves can change the density of the water before and after the wave. The intensity of the parallel light that is projected into the lens after the water with the density variations will change, so we can observe the shock wave on the image.

In the study of this paper, the radius *R*_*max*_ is used as the characteristic parameter of the cavitation bubble when it expands to the maximum volume, *t* represents the time during which the bubbles developed, wherein the subscripts represent different bubble numbers; the distance of two bubbles in the interaction process is represented as *L*. The distance between the bubble and the wall is expressed by the dimensionless parameter *γ*, and its physical meaning is the ratio of the minimum distance from the position of the bubble at the moment of inception to the wall surface *h* and the characteristic parameter of the cavitation bubble *R*_*max*_. *v* represents the velocity of jet, and *V*_*w*_ represents the velocity of shock wave.

## Results and Discussion

### Jets from two cavitation bubbles collapse

**Figure 2:**
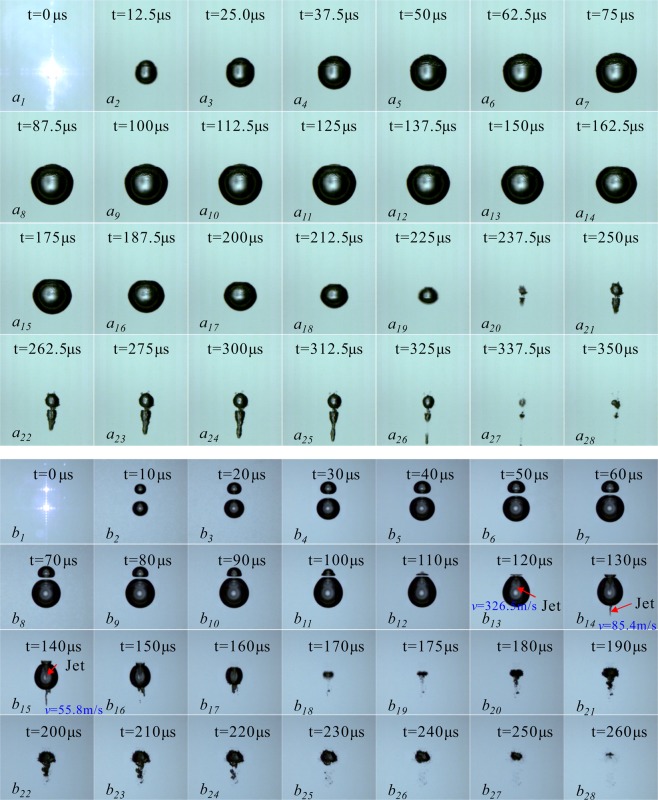
Interactions of two cavitation bubbles generated at the same time with different sizes. (**a**) Frame-rate: 160000 fps, Exposure time: 4.64 μs, Frame width: 5.45 mm; (**b**). Frame-rate: 200000 fps, Exposure time: 2.50 μs, Frame width: 5.45 mm).

Figure 2 is the interaction of two cavitation bubbles of different sizes generated at the same time. In Fig. 2a,b, the frame-rate of 160000 fps and 200000 fps are respectively used in the experiment. The pictures in Fig. 2 are selected from every other one in the high-speed photography sequence. We make the moment when the focused spot penetrates the water and emits dazzling white light as the initial time of the bubble (*t* = 0 μs), and the current evolution time of the cavitation bubble is marked in each picture. In Fig. 2a, the distance between the two cavitation bubbles at the inception moment is very small, about 0.28 mm, the upper one in the image is Bubble_1, and the one at the lower part is Bubble_2, and their maximum characteristic parameters are 0.26 mm and 1.28 mm. In Fig. 2b, the distance between the two cavitation bubbles at the inception moment is about 1.11 mm, the serial numbers of cavitation bubbles are similar to Fig. 2a, the upper one in the image is Bubble_1, and the one at the lower part is Bubble_2. The sizes of the two bubbles developed to the maximum volume are 0.47 mm and 0.89 mm, respectively.

In Fig. 2a, when *t* = 0 μs, the focused spot penetrates the water and emits a dazzling white light. As the pulse of the laser energy ends, the bubble gradually expands outward. When *t* = 75 μs, the Bubble_1 develops to its maximum volume with the maximum radius of 0.26 mm. During the period of 0 μs < *t* < 75 μs, since the distance between the two bubbles is very close, they interact with each other during the expansion. But the energy of Bubble_1 is very small, so that ripples appeared on the surface of the Bubble_2 (on the side close to the surface of the Bubble_1). And as the Bubble_1 expands to its maximum volume, the ripples on the surface of the Bubble_2 gradually advancing toward its far-end surface. During the period of 75 μs < *t* < 137.5 μs, the Bubble_1 enters the contraction phase, while the Bubble_2 continues to expand until it reaches a maximum radius of 1.28 mm. At the same time, the Bubble_1 is gradually swallowed by the Bubble_2 (not completely fused), and the ripples on the surface of the Bubble_2 are gradually weakened. During the period of 137.5 μs < *t* < 237.5 μs, both the Bubble_1 and Bubble_2 contract, and the Bubble_2 shrinks to its minimum volume at *t* = 237.5 μs. It is obvious from Fig. 2 *a*_*20*_ that the minimum volume of Bubble_2 is slightly larger than the one of Bubble_1. During the period of 237.5 μs < *t* < 337.5 μs, both bubbles enter the rebound regeneration stage. It can be clearly seen from the rebound regeneration stage of the two cavitation bubbles that the Bubble_2 shows a pronounced jet under the influence of the Bubble_1 (in the direction away from the Bubble_1), while the Bubble_2 does not show a jet and re-expands only at the original inception position. According to the studies of Obreschkow *et al*.^1^, when *ρgR*_*max*_*/P*_*∞*_ is less than 4*10^−4^, the cavitation bubbles do not form a jet that pierces the far-end surface. Under the experimental conditions of this paper, *ρgR*_*max*_*/P*_*∞*_ = 1.26*10^−4^, which less than 4*10^−4^. However, the Bubble_2 forms a jet that develops away from the Bubble_1 under the influence of Bubble_1. It can be seen that in the free field, a single cavitation bubble is slightly disturbed by the surroundings, which will change the collapse characteristics in its free field.

By adjusting the transmittance of the half-transmitting mirror in the laser-induced cavitation bubble system and the angle of the corresponding focusing mirror to change the relative position and relative size of the two cavitation bubbles. Figure 2b is the interaction between two cavitation bubbles generated at the same time with relatively long distance from each other. The distance between the two at the inception time is 1.11 mm, which is similar to Fig. 2a. When the light spot breaks through the water body, it forms a dazzling white light, which is the time when the bubble is generated, that is, *t* = 0 μs. When the laser pulse energy is over, the bubble gradually expands until *t* = 60 μs when Bubble_1 develops to its maximum volume with a maximum radius of 0.47 mm. And within the period of 0 μs < *t* < 60 μs, the minimum distance of the surfaces of the two bubbles is gradually reduced from 1.11 mm to 0.11 mm. Besides, the surface of the Bubble_1 (the side close to the Bubble_2) takes on a flat bottom form. The Bubble_2 is in an expansion stage at this time, and its surface flat bottom form is not as obvious as Bubble_1. During the period of 60 μs < *t* < 110 μs, the Bubble_1 continues to shrink, while the Bubble_2 continues to expand to the maximum volume, and the distance between the Bubble_1 and the Bubble_2 is further reduced to 0.06 mm, and the adjacent surfaces of the two bubbles exhibit a flat bottom form. In this process, the far-end surface of the Bubble_1 (the surface away from Bubble_2) shrinks faster than the surface near the Bubble_2, which is an asymmetric contraction form. With the strong asymmetric contraction of the Bubble_1, finally Bubble_1 formed a jet that pierced Bubble_2. Since the jet develops inside the cavitation bubble, it moves very fast, the velocity of the jet is about 326.50 m/s obtained by image measurement. When piercing the far-end surface of the bubble, the velocity of the jet decreases sharply to 85.4 m/s due to the retardation of the water. When *t* = 140 μs, the velocity of the jet drops to 55.80 m/s. Within 140 μs < *t* < 175 μs, the Bubble_2 begins to enter the contraction phase. As Bubble_2 gradually shrinks, the jet of the Bubble_1 to it is further developed. It can be seen from Fig. 2 *b*_*16*_ that when the jet piercing the far-end surface of the Bubble_2, not only the velocity gradually decreases, but also the forms changes, bifurcation occurs at the tip of the jet, and finally the jet breaks. During the period of 175 μs < *t* < 260 μs, the two bubbles gradually fuse into one and enter the rebound regeneration stage.

**Figure 3:**
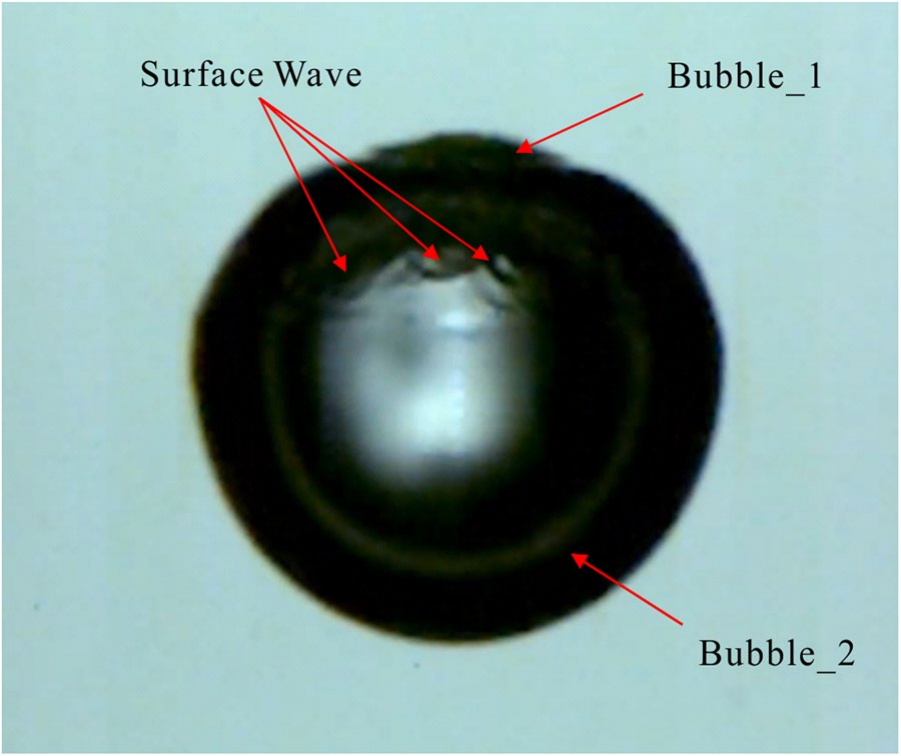
Surface wave of cavitation bubbles.

**Figure 4:**
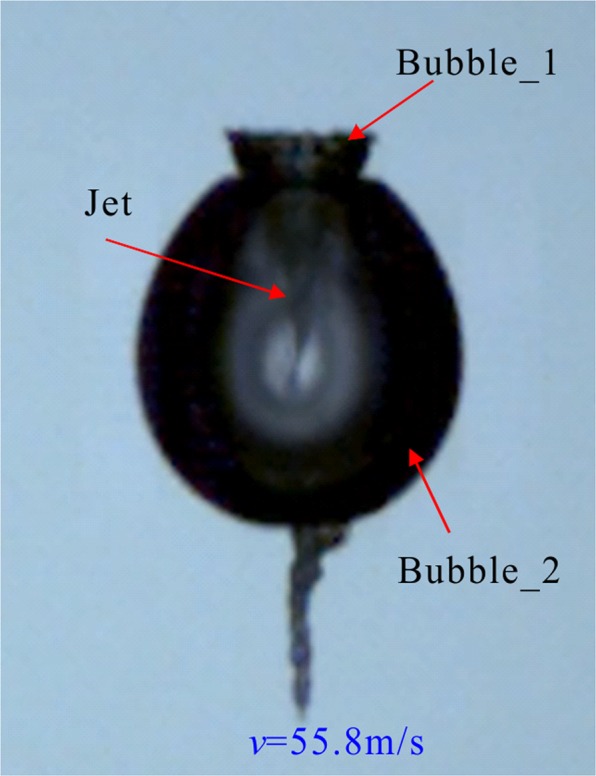
Jet inside the bubble.

Figures 3 and 4 are the surface wave and the internal jet of Fig. 2 (corresponding to *a*_*10*_ and *b*_*14*_ in Fig. 2, respectively). In Fig. 3, since the two bubbles are very close, the Bubble_1 is not synchronized with the expansion-contraction cycle of the Bubble_2 during its development, which leads to surface wave on the larger bubble (Bubble_2), and the development of this surface wave seriously affects the collapse form of the Bubble_2. In Fig. 4, the distance between the two is slightly further, and the Bubble_1 is slightly larger than the Bubble_1 in Fig. 2a, but the expansion-contraction cycles of the two cavitation bubbles are still not synchronized, eventually leading to the appearance of jets. In addition, it is apparent from Fig. 4 that the development of the jet inside the Bubble_1 exhibits a funnel shape, and when the jet pierces the far-end surface of the Bubble_2, a significant discontinuity occurs. From the center of Bubble_1 to the end of the jet, the inside of the Bubble_2 exhibits a funnel shape, and at the end of the jet, the jet width is also tapered, eventually breaking.

Chew^31^ and Rui^32^ studied the interactions of two cavitation bubbles of different sizes through low-voltage discharging method. The two cavitation bubbles described herein are induced by the contactless laser, so there are the following differences: (1) the relative sizes and relative distances of the two bubbles obtained in this paper are far less than the range studied in the literature (and the bubbles are generated at the same time, and there is no phase difference); (2) under the condition of no external interference, we found that when two cavitation bubbles generated at the same time have a big difference in size and the distance between the two bubbles is very close, the small bubble only forms a surface wave towards the big one while the big bubble forms a jet that is far away from the collapse of the small bubble. It should be noted that due to the limitations of the experimental technique, we have not been able to obtain the critical value at which point that the extremely small bubble exerts no influence on the big one.

It can be seen from Figs 3 and 4 that for two cavitation bubbles generated at the same time, as the distance between the initial bubbles and the volume of the smaller bubble gradually increase, the smaller bubbles gradually form surface wave and internal jet for the larger ones, and under the interference of surface waves, larger bubbles eventually form jets away from the smaller ones.

**Figure 5:**
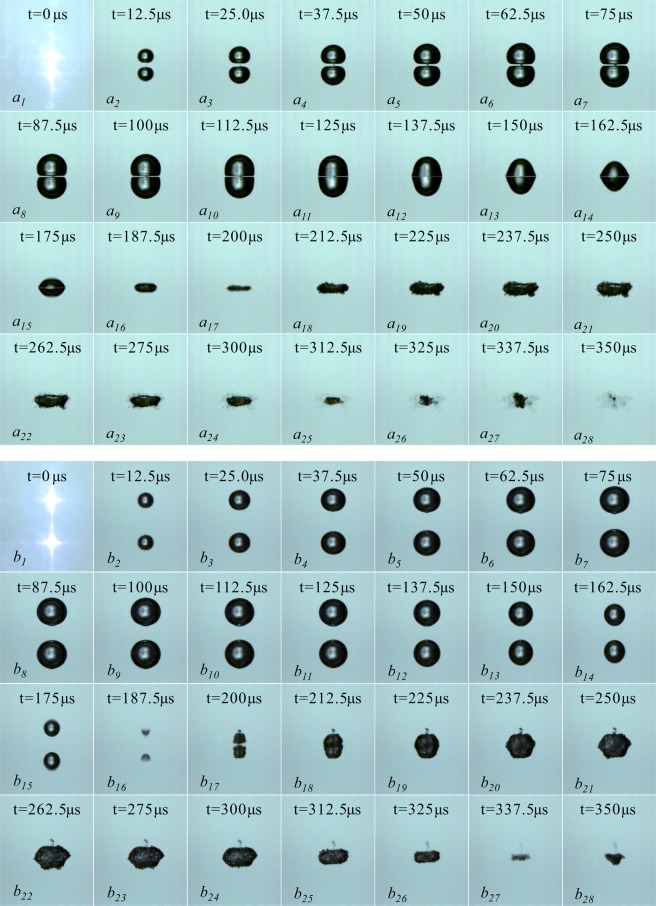
Interactions of two cavitation bubbles generated at the same time with same sizes. (Frame-rate: 160000 fps, Exposure time: 4.64 μs, Frame width: 5.45 mm).

Figure 5 is the interactions of two cavitation bubbles generated at the same time with same sizes. In Fig. 5, the frame-rate of the two groups of experiments is 160000 fps. The pictures in Fig. 5 are selected from every other one in the high-speed photography sequence. We make the moment when the focused spot penetrates the water and emits dazzling white light as the initial time of the bubble (*t* = 0 μs), and the current evolution time of the cavitation bubble is marked in each picture. In Fig. 5a, the distance between the two cavitation bubbles at the inception moment is about 1.23 mm, the upper one in the image is Bubble_1, and the one at the lower part is Bubble_2, and their maximum characteristic parameters are 0.75 mm. In Fig. 5b, the distance between the two cavitation bubbles at the inception moment is about 2.53 mm, the serial numbers of cavitation bubbles are similar to Fig. 5a, the upper one in the image is Bubble_1, and the one at the lower part is Bubble_2. The sizes of the two bubbles developed to the maximum volume are 0.83 mm and 0.84 mm, respectively.

In Fig. 5a, during the period of 0 μs < *t* < 112.5 μs, the two cavitation bubbles expand synchronously to the maximum volume, and during the expansion, the respective central positions remain substantially unchanged, while the adjacent side surfaces gradually becoming planar, and the liquid in the middle of the two bubbles is gradually squeezed by the expansion of them. When expanded to the maximum volume, the middle layer becomes a very thin liquid film, but as can be seen from the image, the two bubbles did not fuse. During the period of 112.5 μs < *t* < 200 μs, the two bubbles gradually shrink. During the shrinking process, the adjacent surfaces of the bubbles still appear planar, while the surfaces away from each other violently shrink toward the center of the respective bubbles. And during the shrinking process, there was no obvious fusion between the two, as well as no jets. During the period of 200 μs < *t* < 312.5 μs, the bubble enters the rebound phase. At this stage, the surface of the bubble appears to be non-smooth. During this rebound expansion and contraction, the two bubbles are finally integrated into one.

In Fig. 5b, the distance between the two cavitation bubbles at the time of inception is 2.53 mm. During the period of 0 μs < *t* < 112.5 μs, the two bubbles expand synchronously to their maximum volume with the maximum radii of 0.83 mm and 0.84 mm, respectively. During the process, the two bubbles are not interfered by each other, and no obvious deformation forms appear on the adjacent sides. During the period of 112.5 μs < *t* < 187.5 μs, the two bubbles enter the contraction phase. During this phase, the adjacent surfaces exhibit a slight mutual attraction effect, and the bubbles take on an ellipsoidal shape, as shown in Fig. 5 *b*_*14*_ and *b*_*15*_. And during the synchronous shrinkage process, the surface contraction speed away from each other is faster than the adjacent sides, and finally the jet emerges when the bubble shrinks to the minimum distance, as shown in Fig. 5 *b*_*16*_. During the period of 187.5 μs < *t* < 337.5 μs, the two bubbles entered the rebound phase. During this phase, both bubbles shoot jets to each other as shown in Fig. 5 *b*_*17*_, and two bubbles are fused at *t* = 212.5 μs. After the fusion, the two bubbles move more vigorously on the upper and lower surfaces, and finally become tabular as in Fig. 5a.

Rui *et al*.^32^ studied the fusion mechanism of two cavitation bubbles near the wall with the low-voltage underwater discharge test method. The influence of the wall on the two-bubble collapse was not covered in this study, but the research case in this paper was similar to the “weak” affected area in the literature. By comparing the experiments in this paper and numerical results in the literature, we can find that although the experimental methods and the sizes of the bubbles are different, the form of the fusion of the two bubbles in this paper is basically consistent with the simulated pattern in the literature. Especially, in the late stage of cavitation bubble collapse, the surface forms of the upper and lower cavitation bubbles are basically symmetrical (see Fig. 5 *a*_*8*_ *~* *a*_*17*_). It can be seen that for two cavitation bubbles of the same size that are generated at the same time, in the case where the distance between the two bubbles is very small, the collapsed form is similar to that of a single cavitation bubble in the boundless domain. Due to the limitations of experimental technique, this paper only qualitatively analyzed the fusion process of two bubbles of the same size. The collapse strength after the fusion of the two cavitation bubbles is often the most concerned issue in practical engineering, which requires more in-depth quantitative researches.

It can be concluded from the analysis of Figs 2~5: (1) If the two cavitation bubbles are synchronously generated but different in size, as the distance between the two cavitation bubbles increases or the maximum radius of the smaller cavitation bubble increases, the effect of the small cavitation bubble on the large one gradually changes from the surface wave phenomenon to internal jet flow that breaks through the large bubble. (2) If the two cavitation bubbles are generated at the same time with same size, as the initial distance of the two cavitation bubbles gradually decreases, the two bubbles are more likely to fuse, and the smaller the distance between the two, the easier it is to fuse.

### Shock Waves from the Collapse of Two Cavitation Bubbles

**Figure 6:**
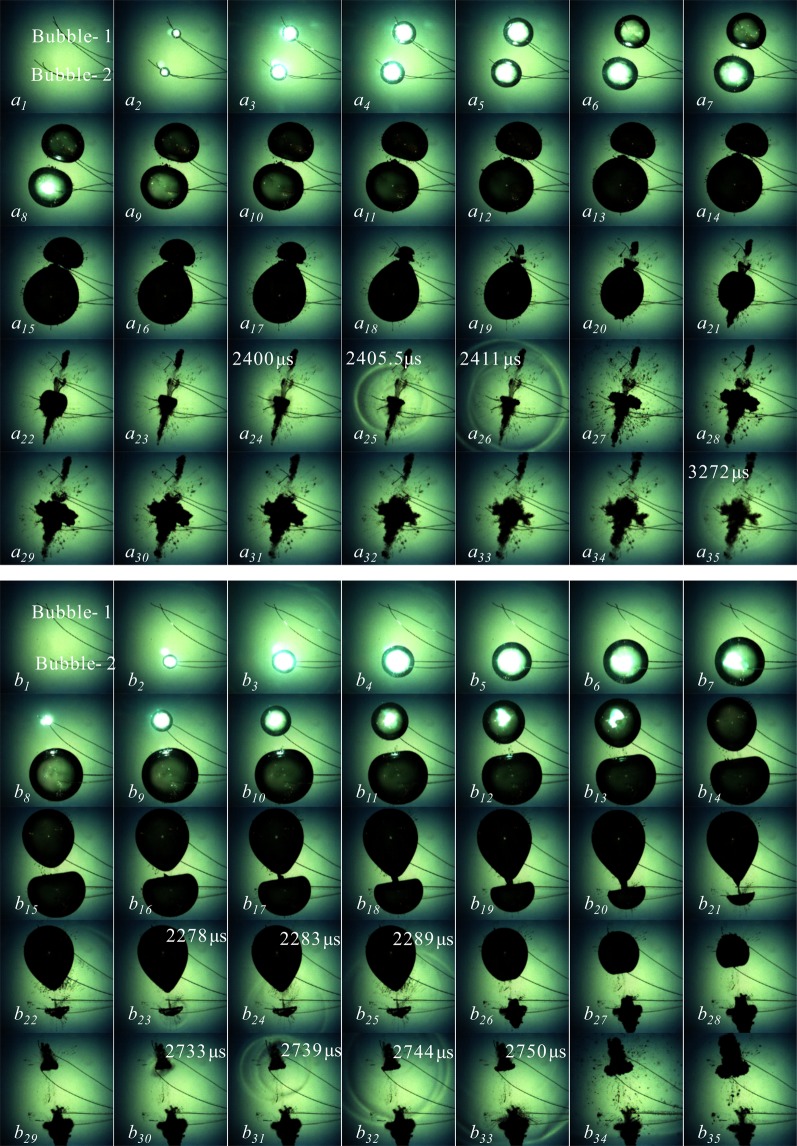
Interactions of two cavitation bubbles. (Frame-rate: 180000 fps, Exposure time: 0.25 μs, Frame width: 37.65 mm).

There are two typical bubble dynamics are presented during the collapse process of cavitation bubbles, namely jets and shock waves. In the previous section, we focused on the jet phenomenon exhibited by the interactions of two laser-induced cavitation bubbles. In this part, underwater low-voltage discharge technology is used to induce two cavitation bubbles, so as to study the differences between shock waves resulting from collapse of two cavitation bubbles and the ones from single cavitation bubble. The differences between the jets resulting from collapse of spark-induced two cavitation bubbles and the ones from laser-induced two cavitation bubbles are also analyzed and compared. Figure 6 is the interaction processes of two cavitation bubbles generated at the same time with different sizes and the interaction processes of two cavitation bubbles generated at different time with different sizes, respectively. The frame-rate of the two groups is 180,000 fps. In order to obtain a clear bubble collapse shock wave, the exposure time is reduced to 0.25 μs during the shooting. In addition to the images of the shock waves, the other images in Fig. 6 are from every 10 pictures in the sequence of the captured images. The time for the appearance of the shock wave has been time stamped in Fig. 6. In Fig. 6, the bubble on the upper part of each image is Bubble_1, and the bubble on the lower part is Bubble_2.

Figure 6a is the interaction between two cavitation bubbles generated at the same time with different sizes. The distance between the two at the inception time is 16.19 mm. Both the cavitation bubbles experienced the expansion and contraction phases during the period of 0 μs < *t* < 2411 μs. During this phase, the two bubbles expand synchronously, but since the energy obtained by the Bubble_1 in the initial stage is smaller than that of the Bubble_2, the Bubble_1 expands to the maximum radius first than the Bubble_2, as shown in Fig. 6 *a*_*11*_. The Bubble_2 reached the maximum volume 578 μs later than Bubble_1, as shown in Fig. 6 *a*_*15*_. During the expansion of the Bubble_1, the surface near Bubble_2 is flat. Subsequently, the two bubbles are in the stage of contraction and collapse. In this stage, due to the small volume of the Bubble_1, the surface away from Bubble_2 shrinks faster. Finally, when Bubble_1 shrinks to its minimum volume, a jet that penetrates the Bubble_2 is formed as shown in Fig. 6 *a*_*21*_. Rui^32^ studied the characteristics of the micro-jets formed when the two cavitation bubbles collapse with the same test method as this paper. Therefore, this paper mainly discusses the shock waves from the collapse of two cavitation bubbles. To capture the shock wave, the exposure time must be reduced to 0.25 μs. Therefore, the development of the jet inside Bubble_2 is not observed in the image, but it is apparent from Fig. 6 *a*_*21*_ the jet is developing very fast and has penetrated the far-end surface of the Bubble_2. For *a*_*24*_, *a*_*25*_ and *a*_*26*_ in Fig. 6a, this shock wave should be induced from the collapse of the Bubble_2, because the Bubble_1 has formed a breakdown jet before the shock wave appears. It can be seen from the waveform structure of the wave that the development of the shock wave is not completed once, but the second wave has been developed in a very short time after the first shock wave has been developed, as shown in Fig. 6 *a*_*25*_ and *a*_*26*_.

Figure 6b is the interaction between two cavitation bubbles generated at different time with different sizes. The bubble center distance is about 18.06 mm, and the Bubble_2 is generated 728 μs earlier than the Bubble_1. When the Bubble_2 develops to the maximum volume, the Bubble_1 is generated. In this stage, the Bubble_2 is spherically expanded, but when the Bubble_1 is generated, the surface of the Bubble_2 (near the side of the Bubble_1) gradually shows a flat bottom shape, as shown in Fig. 6 *b*_*8*_ ~ *b*_*13*_. During the phase of *b*_*9*_ ~ *b*_*21*_, Bubble_1 continues to expand, while the Bubble_2 enters the contraction phase. At the time of *b*_*21*_, the Bubble_1 has developed to the maximum volume, and the Bubble_2 has not contracted to the minimum volume. At the time of *b*_*23*_, the Bubble_2 shrinks to the minimum volume. It can be seen from the image that the two bubbles are not fusing. The Bubble_2 exhibits a hemispherical-shape contraction, and the surface on the side far from the Bubble_1 contracts faster. Finally, at the time of *b*_*23*_, the Bubble_2 has a collapse shock wave, and the shock wave does not affect the shape of the Bubble_1 during the outward development. After that, the Bubble_1 continues to shrink and shrinks as shown in Fig. 6 *b*_*30*_. And a significant shock wave appears, and the development of which does not have a significant effect on the Bubble_2 in the rebound phase.

**Figure 7:**
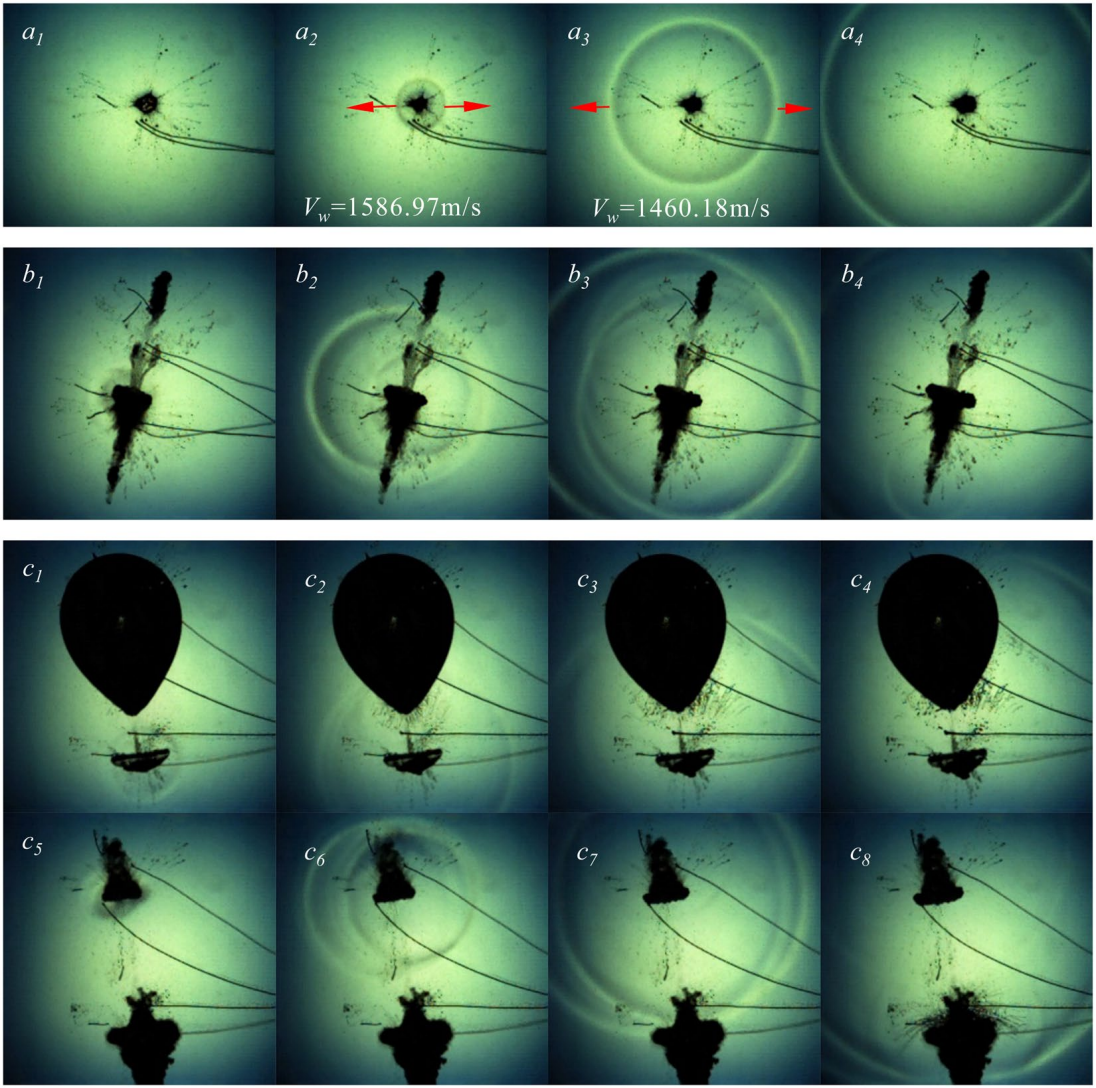
Shock wave induced by collapse of two cavitation bubbles.

Figure 7 is a detailed view of the bubble collapse shock wave. Figure 7a is the shape of the shock wave when a single bubble collapses. Figure 7b is the shock wave resulting from collapse of two cavitation bubbles generated at the same time. Figure 7c is the shock wave resulting from collapse of two cavitation bubbles generated at different time, where Fig. 7b,c are from Fig. 6a,b, respectively. It can be clearly seen from Fig. 7a that a single bubble does not form a jet during collapse, the shock wave shape is very close to a sphere, and the outward spread is very fast, and the velocity is gradually attenuated, which reduced from 1586.97 m/s to 1460.18 m/s in 5.56 μs. We used the frame-rate of 180000 fps in the experiment, and only three pictures were captured during the entire bubble collapse process.

Figure 7b is a shock wave resulting from the collapse of two cavitation bubbles generated at the same time. Under this condition, the collapse shock wave of the bubble comes from the bubble of larger size. It can be seen from Fig. 7b that the shock wave resulting from the collapse of them is completely different from that of the single bubble collapse. The main manifestation is that the bubble collapse shock wave is not released once, but following waves appears successively, and the wave superposition appears in some areas. As shown in Fig. 7 *b*_*3*_, *c* is a shock wave resulting from the collapse of two cavitation bubbles generated at different time, in which Fig. 7 *c*_*1*_ ~ *c*_*4*_ is the earlier cavitation collapse shock wave, and Fig. 7 *c*_*5*_ ~ *c*_*8*_ is the later cavitation collapse shock wave. The collapse shock wave of the earlier cavitation bubbles in Fig. 7c develops independently and does not affect the form of the late-generated bubbles. Its form and development are similar to those of a single bubble collapse. For collapse shock wave of the later cavitation bubbles, the wave is not released once, but two shock waves appear in a short period of time, and in the process of outward development, wave superposition occurred in some areas. As shown in the wave shape in the lower part of Fig. 7 *c*_*8*_.

It can be clearly seen from Figs 6 and 7 that the initial moment of the cavitation bubbles not only affects the jet, but also has a significant effect on the structure of the collapse shock wave. For cavitation bubbles generated at the same time with different size, the shock wave is mainly caused by the large bubble collapse. For cavitation bubbles generated at different time, the two bubbles have shock waves from the collapse one after another. The shock waves in both cases are completely different from the single spherical shock wave of the single bubble collapse, there are continuous waves appear in a short time. And due to different speeds, these may have waves superposition in some areas.

### Characteristics of near wall two cavitation bubbles collapse

**Figure 8:**
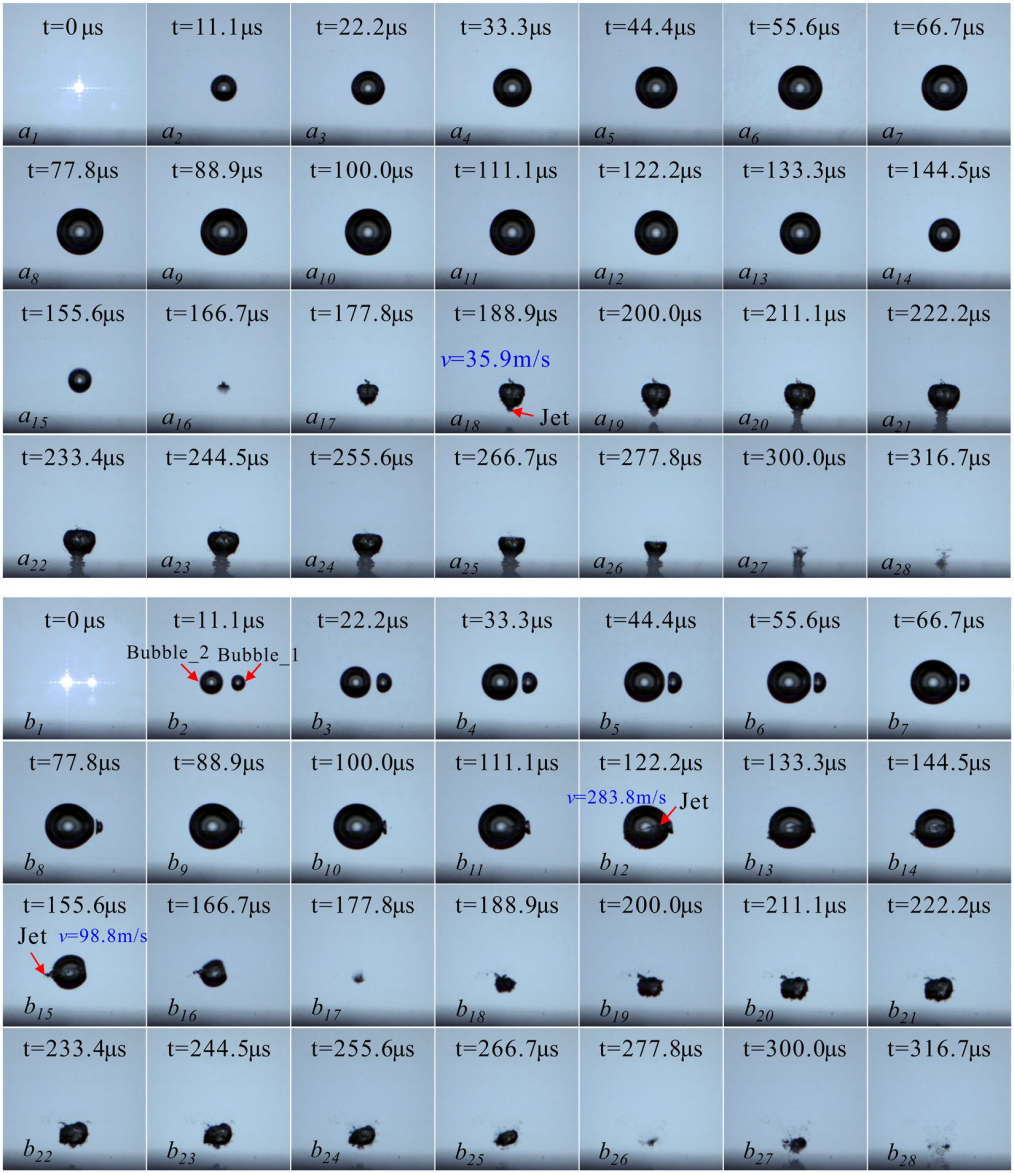
Impact Processes on Wall Surfaces by Collapses of Single and Two Cavitation Bubbles on the Near Wall. (Frame-rate: 180000 fps, Exposure time: 2.50 μs, Frame width: 5.45 mm).

For the jet and shock wave phenomena that occur in multiple bubble collapses, the above analysis is carried out by using different experimental systems. However, for the utilization and prevention of multi-cavitation interactions, it is necessary to consider the influence of boundary conditions around multi-cavitation bubbles. This section only studies the collapse modes of the two bubble center lines parallel to the wall. Figure 8 is the collapse on the near wall of two cavitation bubbles of same inception time. Figure 8a is the evolution of one single cavitation bubble. As a comparative experiment, Fig. 8b is the collapse process of two cavitation bubbles near wall.

It can be clearly seen from Fig. 8a that the cavitation bubble does not directly form the jet that strike the wall surface during the first expansion and collapse process, and the jets are formed after the resilience of the cavitation rebound, as shown in Fig. 8 *a*_*18*_. This is mainly because the cavitation bubble is far away from the wall surface at the time of inception, and its characteristic parameter *γ* = 1.90. During the second expansion and collapse of the cavitation bubble, the cavitation bubble moves toward the wall surface quickly. For Fig. 8b, the maximum radius of the larger cavitation bubble is *R*_*max*_ = 0.86 mm, which is basically the same as the size of Fig. 8a. However, due to the presence of a small cavitation bubble around, the larger one is penetrated in its first stage of expansion-contraction by the jet from the small bubble, which disturbs the law of the development of the large cavitation itself to the wall surface. The main manifestations are: (1) under the condition of a single cavitation bubble, the bubble shrinks to the minimum volume. The time when the bubble shrinks to the minimum volume is 166.7 μs, and when there is a smaller cavitation bubble, the time when it shrinks to the minimum volume increases to 177.8 μs. (2) Under the small cavitation disturbance, the asymmetric shrinkage pattern of the bubble to the wall changes. Under the condition of a single bubble, the surface of the bubble away from the wall surface shrinks faster. And when there is disturbance of a small cavitation bubble, due to the small bubble forms a jet that impacts the large cavitation bubble, so that the shape development of the large one is no longer asymmetrical, as shown in Fig. 8 *a*_*15*_ and *b*_*16*_. (3) In the rebound regeneration stage, under the small cavitation bubble disturbance, the cavitation bubble does not form a jet directly impacting the wall surface, but the whole body moves rapidly toward the wall surface.

Chew *et al*.^25^ and Rui *et al*.^32^ studied the interaction of two cavitation bubbles near the wall by the underwater low-voltage discharge method, in which Chew *et al*.^25^ studied the state of the two bubble center line parallel to the wall and Rui *et al*.^32^ studied the state of the two bubble center line perpendicular to the wall. Chew *et al*.^25^ obtained a method of predicting the micro-jets of the two bubbles near the wall by judging the migration direction of the cavitation bubble. In the study of this paper, the details of the internal jet of the small cavitation bubble on the large one and those of the small cavitation bubble suppressing the formation of the micro-jet by the large one to the wall surface can be clearly seen. The literature^32^ divided the interactions of the two bubbles near the wall into three parts according to the bubble-wall distance, and Bubble_1 and Bubble_2 in Fig. 8b of this paper belong to the “weak interaction” zone and the “intermediate interaction” zone described in the literature. Internal jets also appear in the literature. The two bubble center line is perpendicular to the wall surface in the literature while the two bubble center line is parallel to the wall surface in this paper. By comparison, it is found that the formation mechanism of the internal jet in the literature is caused by the existence of the wall surface. However, the internal jets in this paper are caused by bubble-bubble interactions. For the interaction of two cavitation bubbles in the near-wall area, the jet inhibition influence of small cavitation bubbles on the larger ones requires more in-depth quantitative researches. This paper aims to qualitatively discuss the physical process of this inhibition.

## Conclusions

By respectively inducing two cavitation bubbles through laser and underwater low-voltage discharge, this paper tested the jet and shock wave during the collapse of the two cavitation bubbles with different time of inception, and the following conclusions are obtained:

1. If the two cavitation bubbles are synchronously generated but in different size, as the distance between the two cavitation bubbles increases or the maximum radius of the smaller cavitation bubble increases, the effect of the small cavitation bubble on the large one gradually changes from the surface wave phenomenon to jet that breaks through the large bubble. If the two cavitation bubbles are generated at the same time with same size, as the initial distance of the two cavitation bubbles gradually decreases, the two bubbles are more likely to form a face to face collapses, and the smaller the distance between the two, the easier it is to fuse. When the two bubble center lines are parallel to the wall surface, the above-mentioned jet will suppress the formation of the jet to the wall surface when the large cavitation collapses.
2. The initial moment of the cavitation bubble also has a significant effect on the structure of the collapse shock wave. The collapse shock wave is different from the spherical shock wave of the single cavitation bubble collapse in the free field. Specific manifestation is as follows: for two cavitation bubbles of different sizes formed synchronously, the development of the shock wave in the collapse is dominated by the larger one. For cavitation bubbles generated at different time, they have shock waves successively in the collapses. In both cases, the shock waves are not released once, but a continuous number of waves in a short time. Due to different speeds, these waves may overlap in some areas of the space.

The conclusions obtained from the two cavitation bubble interactions (surface wave and internal jet) induced with the interference-free method and the shock wave test during the two cavitation bubble collapse induced with the underwater low-voltage discharge method have potential value for the anti-corrosion design and the corrosion resistance of hydraulic mechanical blades in hydraulic engineering.
